# The development of indicator measure for monitoring the quality of patient-centered care in China’s tertiary hospitals

**DOI:** 10.1371/journal.pone.0205489

**Published:** 2018-10-11

**Authors:** Huixuan Zhou, Ge Bai, Jiechun Gao, Yinan Zhou, Emily Ma, Linlin Hu, Guangyu Hu, Pengyu Zhao, Feng Jiang, Li Luo, Yuanli Liu

**Affiliations:** 1 School of Public Health, Chinese Academy of Medical Sciences & Peking Union Medical College, Beijing, P.R. China; 2 Hospital Management Institute, Fudan University, Shanghai, P.R. China; University of Malta Faculty of Health Sciences, MALTA

## Abstract

**Objective:**

To develop a set of structure and process indicators to evaluate tertiary hospitals’ performance in the Healthcare Improvement Initiative, a national program with a goal to improve quality of patient-centered care.

**Methods:**

A modified Delphi technique, including literature review, multidisciplinary panel meeting and anonymous rating, was used to generate a set of indicators. A practice test involving both general and special hospitals was conducted to ensure the feasibility of data collection for these indicators.

**Results:**

62 indicators were generated by literature review. The panel review procedure involving 39 panelists with diverse backgrounds resulted in a total of 59 indicators, which included 40 qualitative indicators and 19 quantitative indicators. In the practice test, six quantitative indicators were found unfeasible. According to the suggestion of the experts in the hospital evaluation committee, three of those indicators were kept by adjusting their data collection methods, while other three ones were discarded.

**Discussion:**

A set of 56 structure and process indicators was developed to evaluate hospitals’ performance in the implementation of the Healthcare Improvement Initiative, which could be used in both general and special tertiary hospitals. Results of the indicator measurement could present a panorama of the quality of patient-centered care in tertiary hospitals nation-wide, and inform health administrators of the ways to attain the goal of the Initiative.

## Introduction

Patient-centered care (PCC) can be commonly understood as healthcare which can cater to patient needs [1]. The elements which constitute PCC may vary among different professional groups, while the core components of PCC can be identified as (a) patient participation and involvement (e.g., customized care plan, addressing patient’ emotional needs as well as physical needs), (b) patient- doctor relationship (e.g., open communication of information, personal qualities of health professionals, such as appropriate skills, good etiquette, respectful and welcoming attitudes), and (c) the setting where healthcare is delivered (e.g., appropriate treatment time, adequate staff and friendly facilities) [2, 3].

As PCC has been increasingly recognized by health administrators across the world [4–6], the China’s government has become interested in improving quality of PCC especially in the tertiary public hospitals. Tertiary public hospitals account for 7.66% of the all-type health facilities in China, yet provide 42.50% of the inpatient care and 48.70% of the outpatient services [7]. Some patient surveys find room for service improvement in these overcrowded tertiary hospitals. For instance, the fifth national health survey indicates that 34.4% of patients are unsatisfied with outpatient environment [8]. Specifically, windows for registration and payment are concentrated in the hall of outpatient building, and online systems for registration, payment and inquiry are underdeveloped in most tertiary hospitals, which lead to long waiting time and overcrowding of patients in peak visiting periods [9]. Patients averagely spend 2.5 to 8 hours in the hospital for a consulting lasting 15–20 minutes [10]. Moreover, surveys among tertiary hospitals in -Beijing and Shanghai indicate that more than 70% of patients are dissatisfied with toilet hygiene because of smelly odor and filth in urinals[11]. More than 30% of patients are not satisfied with inpatient care mainly because of process inefficiency, concerns about quality and safety and attitudes of medical staff [8].

To target concerns of patient, the Ministry of Health (MoH, renamed as National Health and Family Planning Commission in 2014) came up with a new vision for improving healthcare, and implementing the Healthcare Improvement Initiative (Initiative for short) in January of 2015 [12]. The overall goal of this program is to make healthcare more patient-focused and improve patients’ experience and satisfaction. The Initiative encompasses 29 actions falling under nine objectives, which include, within a three-year time frame, (1) optimize the layout of the facilities and build a friendly service environment, (2) promote utilization of medical appointment services and guide patient flow, (3) improve service efficiency by rational allocation of resources, (4) take advantage of information technology to improve patient’ experience, (5) improve process reengineering and accommodation in inpatient department, (6) continuously improve quality of nursing care and enhance nursing workforce, (7) ensure patient safety by adoption of standard operating procedures, (8) strengthen humanistic care and provide social work services, (9) harmonize doctor-patient relationship and reduce medical disputes [12]. The objectives and actions of the Initiative were set by the MoH by canvassing the views of health administrators and public opinions in social media [13].

In order to assess the performance of tertiary public hospitals in the Initiative, the MoH required our research team to develop a set of indicators to measure whether those actions have been implemented. Although indicators for accreditation of tertiary hospitals has been constructed by the MoH since 2010[14], and some studies have developed indicator measurement from specific aspects (such as safety culture in radiological department, safety of nursing care, and quality of impatient care in general hospitals) [15–17], a set of indicators to comprehensively measure the quality of PCC in different types of tertiary hospitals is in demand. The development of indicators for the Initiative is supposed to focus on the quality of PCC, and evaluate the performance of both general and special tertiary hospitals in this national program in a more specific way.

According to the commonly used Donabedian Model, quality of healthcare can be measured by three types of indicators: structure, process and outcome indicators [18]. According to the objectives of the Initiative, structure indicators measure the settings of hospitals in program implementation, for instance, whether the hospital has regulations and facilities to support quality improvement of patient-centered care; process indicators assess the degree to which actions are being effectively implemented; outcome measurement, such as patient experience or satisfaction, can be taken as ultimate validator of hospitals’ performance, which could be measured by patient survey about waiting time, utilization of online services and their subjective feeling of medical environment, process as well as doctor-patient relationships [2].

This paper describes the development and practice test of structure and process indicators from the perspective of health organizations [19]. The outcome measurement from the perspective of patient, as per MOH’s requirement, will be developed by other studies in the form of questionnaire survey among patients.

## Methods

A modified Delphi technique (RAND/UCLA Appropriateness Method), comprised of literature review, multidisciplinary panel meeting and two rounds of anonymous rating [20], was used in our study. This method has been commonly used to develop quality indicators in developed countries [19, 21–24]. Feasibility of proposed indicators was verified by practice test in pilot hospitals.

When evaluating the appropriateness of candidate indicators, criteria as follows were considered by research team and panelists [21, 25, 26]:

Validity. An adequate indicator must have empirical rationale for its relevance to quality of patient-centered care.
Utility. The structure and process indicators can be improved through changes in hospital administration or the way of service delivery, according to the measures of the Initiative.
Feasibility. The data for indicator assessment can be extracted from hospital information systems, or can be explicitly observed in survey.

### Development of structure and process indicators by the modified Delphi technique

#### Literature review

**Fig 1:**
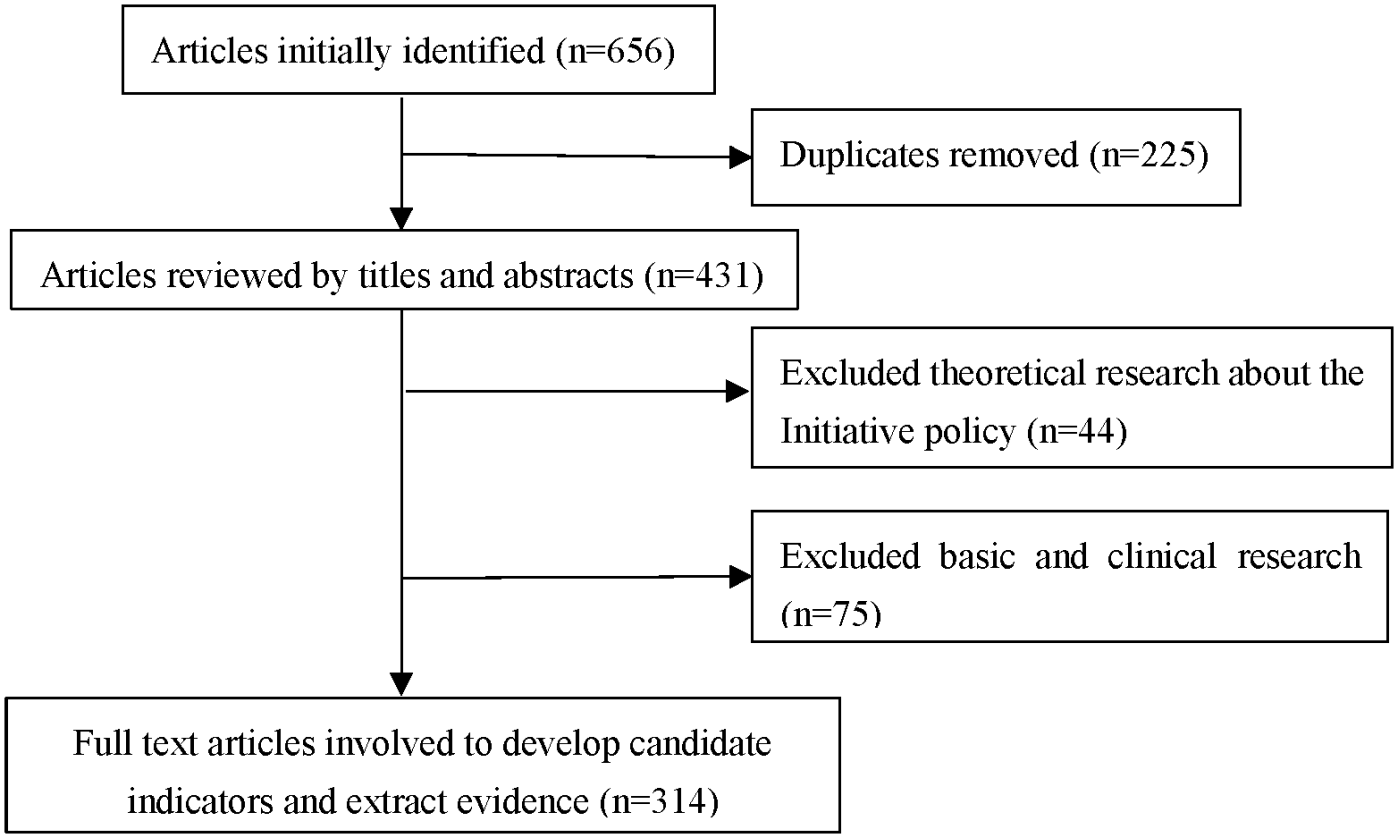
Flowchart of article screening.

Literature review was conducted to generate candidate indicators and their description for panel review. We searched articles in China National Knowledge Infrastructure database and PubMed database in June 2015, with search terms ‘Healthcare Improvement Initiative’, ‘service environment’, ‘medical appointment’, ‘allocation of resources’, ‘day surgery’, ‘emergency priority’, ‘health information technology’, ‘inpatient care’, ‘patient safety’, ‘humanistic care’, ‘social work’, ‘doctor-patient relationships’, ‘quality indicators’ and ‘hospital accreditation’. Those search terms were developed according to the objectives and actions of the Initiative. A total of 656 articles were initially identified, after article screening 314 articles of full text addressing healthcare quality control were finally selected. Fig 1 illustrates the inclusion and exclusion of the articles in literature review. We not only developed candidate indicators by article review, but also extracted some evidence from articles describing the process, results and problems in delivering patient-centered care. The evidence could support the validity and utility of some indicators.

In addition, we also reviewed the indicators and standards in three quality measurement systems: the China Tertiary Hospitals Accreditation (THA), the US Joint Commission International (JCI), and the German Cooperation for Transparency and Quality in Healthcare (KTQ) [14, 27–29]. To our knowledge, these systems included some structure and process indicators related to patient-centered care, which could be measured at organizational level. According to the content of the Initiative policy, we could extract some candidate indicators from these systems to meet our research aim.

GB and HZ extracted indicators and evidence, integrated information, and finally formed a document for candidate indicators, which included the definition, algorithm for quantitative indicator, standards for qualitative indicators, and data collection methods (such as survey, administrative data and medical records) for each of the indicators [25]. The origin of candidate indicators (from article review or indicator systems of THA, JCI and KTQ) were also marked. Candidate indicators were categorized into 29 specific actions falling under nine objectives of the Initiative. We coded the candidate indicators in a hierarchical format. For instance, ‘1.2.3’ indicates that this indicator is the third one assessing the implementation of the second action, which belongs to the first objective of the Initiative.

#### Multidisciplinary panel

We selected the panelists considering their experiences in hospital management or clinical practice as well as their geographical diversity. Six universities, five research institutes and six tertiary hospitals located in different areas of China, were required to nominate panelists to participate in the panel review. Ultimately, 39 panelists with multidisciplinary backgrounds (16 professors major in health management and policy, 10 physicians and 13 hospital managers) consented to participate in two rounds of anonymous rating by Email and a face-to-face panel meeting between two rating procedures in Shanghai.

#### First round rating

Documents including the information of candidate indicators and rating instruction were sent to the panelists by Email. According to the RAND/UCLA Appropriateness Method [20], the panelists were asked to rate each indicators by circling a number from 1 (not at all appropriate to assess the implementation of the Initiative) to 9 (very appropriate) in a questionnaire, and they could add new indicators in the questionnaire. Indicators were selected if the median scores were 7, 8 or 9 without disagreement (1/3 or more ratings in both 1–3 or 7–9 regions). Indicators with the median scores of 1–3 without disagreement were discarded. Indicators with the median scores of 4–6 or with disagreement were involved in the agenda of next step panel meeting.

#### Panel meeting

In the face-to-face panel meeting, the panelists expressed their opinions of the indicators on which they did not reach consensus, rephrased the description of some indicators and discussed the new added ones.

#### Second round rating

According to the opinions of panelists, we compiled all remained indicators with their description (rephrased if necessary), and sent them to the panelists by Email. The panelists rated these indicators in a questionnaire with the same format as the first-round rating. Indicators were accepted if the median rating scores were 7, 8 or 9 without disagreement. Indicators with the median scores of 1–6 were discarded.

### Practice test

#### Settings

Given these indicators will come into use in both general and special tertiary hospitals, we conveniently sampled four tertiary hospitals with different types in Jiangsu Province (a general hospital, a cancer hospital, a maternity hospital and a hospital of Traditional Chinese Medicine) to verify the feasibility of proposed indicators, and test the data resources and data collection methods for these indicators [30].

#### Data collection

In order to efficiently and precisely evaluate tertiary hospitals’ performance, we invited nine experts to form an evaluation committee, and divided these experts into three groups depending on their backgrounds to share the work of data collection. The first group comprised of three health administrators from Health Authority of the MoH, and was responsible for collecting information for the indicators corresponding to the objectives 1, 8 and 9, which were mainly related to clinical environment, humanistic care and doctor-patient relationships. The second group including three chief physicians from a tertiary hospital not involved in the practice test, took charge of data collection for indicators falling under the objectives 3, 6 and 7, which were related to allocation of medical resources, quality of nursing care and patient safety. The third group involving three hospital managers from the same hospital, took responsibility of collecting data for indicators corresponding to the objectives 2, 4 and 5, which were related to medical appointment, information technology and logistics of impatient care.

Each expert was trained with data collection methods (e.g., to observe hospital environment, review hospital documents in survey and abstract information from administrative data or medical records). We asked experts in each group to supervise each other and reach consensus on result of each indicator. Each group was required to note results of qualitative indicators and original data of quantitative indicators in a questionnaire.

#### Feasibility assessment

A specific indicator was considered ‘unfeasible’, if the information for its measurement could not be obtained by its data collection method [25], which resulted in missing data in the questionnaire. We discarded unfeasible indicators or adjusted their data collection methods according to the suggestions of experts.

## Results

**Fig 2:**
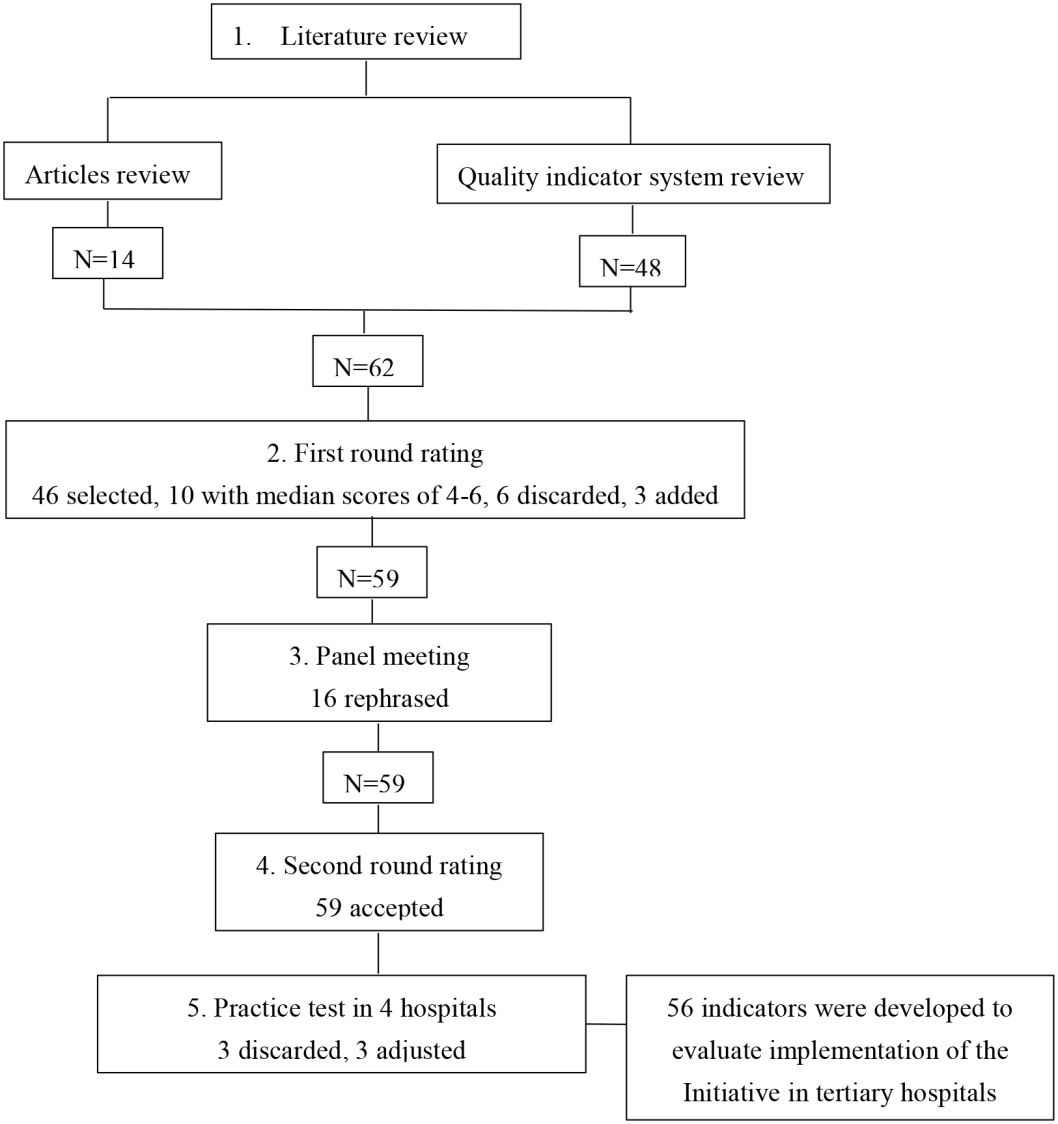
Flowchart of indicator development and resulting inclusion and exclusion of indicators.

Fig 2 illustrates the inclusion and exclusion of indicators in each step of the modified Delphi technique and practice test.

### Structure and process indicators for the Initiative implementation

#### Selection of indicators from literature

We developed 14 indicators by article review. Quality indicator systems review yielded 48 indicators. Specifically, 35 indicators were derived from China Criteria for Accreditation of Tertiary Hospitals, 11 indicators were derived from JCI and 2 indicators were derived from KTQ. In total, 62 indicators were involved in the panel review procedure. Moreover, we also extracted some evidence by article review to support the validity and utility of some indicators. For instance, Ji’ research demonstrates that patients spend most of their time on waiting for consulting, especially for patients with common ailments, thereby suggests that tertiary hospitals should distribute consulting rooms according to patients’ need and arrange more rooms for department treating common diseases [9]. Depending on evidence like that, indicator “1.1.1 Improve the layout of consulting rooms” could be considered valid, and to arrange consulting rooms according to outpatient flow analysis was supposed to be an effective way to reduce waiting time for patients.

#### First round rating

All 39 panelists responded the rating questionnaire by Email in four weeks. The panel reached consensus on 46 indicators (the median scores were 7, 8, 9 without disagreement), rejected six indicators (the median scores were 1, 2, 3 without disagreement) and suggested three new indicators. Ten indicators got median scores of 4–6 without disagreement, and required panelists’ opinions on their inclusion, adjustment or exclusion in the panel meeting. Table 1 presents these indicators with the corresponding objectives and actions, as well as their origins (article review, indicator systems of THA, JCI and KTQ, added by panelists), types (structure or process indicators) and median scores. The details of rating which were used to calculate median score and disagreement could be found in S1 Dataset.

**Table 1:** Candidate indicators in first round rating.

Objective	Action	Indicator		Origin	Type	Med-ian
1. Optimize the layout of the facilities and build a friendly service environment	1.1 Improve the layout of facilities to reduce waiting time.	1.1.1	Improve the layout of consulting rooms.	THA	S	7
		1.1.2*	Concentrate the regions for consulting, examination, payment and pharmacy.	THA	S	3
		1.1.3	Set up adequate registration and payment windows.	THA	S	7
	1.2 Maintain a tidy environment	1.2.1	Build hygiene maintenance system.	THA	S	7
		1.2.2	Keep toilets hygiene.	THA	S	8
		1.2.3	Enforce smoking ban.	THA	S	6
	1.3 Construct cautionary infrastructure	1.3.1	Present direction signs to guide patients.	THA	S	6
		1.3.2	Provide safety alarm facilities.	JCI	S	7
	1.4 Construct user-friendly public facilities	1.4.1*	Set up guide station to provide counselling service.	THA	S	3
		1.4.2	Provide barrier-free facilities.	AR	S	7
		1.4.3	Offer radiation-free zones.	JCI	S	7
2. Promote utilization of medical appointment services and guide patient flow	2.1 Promote appointment-booking service	2.1.1	Increase appointment-booking rate.	THA	S	8
		2.1.2	Offer privilege and facilities to patients who use appointment service.	THA	S	7
		2.1.3	Increase the rate of appointment-booking for subsequent visit.	THA	P	6
		2.1.4	Increase the rate of appointment-booking for subsequent oral treatment.	THA	P	6
		2.1.5	Increase the rate of appointment-booking for subsequent prenatal examination.	THA	P	8
	2.2 Push forth dual-referrals	2.2.1	Build referral system with secondary hospitals and (or) community health centers.	THA	S	7
	2.3 Allocate time slots for reservation	2.3.1	Offer time slots for examination reservation to inpatients.	AR	S	7
		2.3.2	Offer time slots for consulting reservation to outpatients.	AR	S	8
3. Improve service efficiency by rational allocation of resources	3.1 Appropriately distribute resource	3.1.1	Arrange adequate number of physicians to meet outpatients' need.	THA	S	8
		3.1.2	Provide clinical examination to ED patients in efficiency.	THA	S	8
		3.1.3	Provide biochemical examination and immunologic test to ED patients in efficiency.	THA	S	8
	3.2 Push forth day surgery	3.2.1	Promote day surgery.	AR	S	6
	3.3 Bolster emergency department staffing	3.3.1	Connect ED treatment with pre-hospital care.	THA	S	7
		3.3.2	Ensure the amount of ED physicians.	THA	S	7
		3.3.3	Ensure the amount of ED nurses.	THA	S	5
	3.4 Improve treatment for critical ill	3.4.1	Open green channels in ED.	THA	S	6
		3.4.2	Implement triage in ED.	THA	S	5
4. Improve service efficiency by rational allocation of resources	4.1 Strengthen information guidance	4.1.1	Provide reminder service by Apps.	THA	S	7
		4.1.2	Provide appointment-booking service by Apps.	THA	S	8
		4.1.3	Provide payment service by Apps.	THA	S	7
	4.2 Strengthen information management	4.2.1	Use IT to manage medical records.	AR	S	7
		N/A	Equip pharmacy with automation.	Added	S	N/A
	4.3 Promote inquiry service	4.3.1	Provide facilities for self-help inquiry.	AR	S	8
5. Improve process reengineering and accommodation in inpatient department	5.1 Enhance hospitalization process	5.1.1	Offer admission and discharge instruction to inpatients.	JCI	S	8
		5.1.2	Share patients' information in hospital transfer.	JCI	S	7
	5.2 Improve hospital living conditions	5.2.1	Seriously manage ward visiting.	JCI	S	7
		5.2.2	Offer accompany service to handicap patients.	JCI	S	8
		5.2.3	Offer nutrition service to improve inpatient's diet.	JCI	S	7
	5.3 Develop patient follow-up	5.3.1	Follow up discharged patients according to doctors' advice.	THA	S	7
6. Continuously improve quality of nursing care and enhance nursing workforce	6.1 Bolster nursing staff	6.1.1	Number of nurses in clinical nursing post.	AR	S	7
		6.1.2	Number of nurses to meet inpatients' need.	AR	S	7
		6.1.3	Number of nurses in ICU	AR	S	7
		N/A	Number of nurses in NICU	Added	S	N/A
	6.2 Consolidate quality care	6.2.1	Provide quality nursing care to inpatients.	AR	P	7
		6.2.2	Implement quality nursing care program in wards.	THA	P	7
7. Ensure patient safety by adoption of standard operating procedures	7.1 Consolidate patient safety	7.1.1	Mark surgical site.	JCI	P	8
		7.1.2	Promote inpatient identification.	JCI	P	8
		7.1.3	Manage hand hygiene of medical staff.	KTQ	P	6
		7.1.4	Reduce patient falls.	JCI	P	7
		7.1.5*	Establish ethics committee to approve the use of innovative medical technology.	THA	S	3
		7.1.6*	Monitor patient safety by indicators based on disease groups.	THA	P	3
	7.2 Develop clinical pathways	7.2.1	Follow clinical pathways to manage inpatients.	THA	P	7
	7.3 Strengthen appropriate medication	7.3.1	Control frequency of antibiotic use over inpatient treatment.	AR	P	7
		7.3.2	Control AUD over inpatient treatment.	AR	P	8
	7.4 provide transparent charge service	7.4.1	Release pricing information.	THA	P	7
		N/A	Expand pay per disease payment system.	Added	P	N/A
8. Strengthen humanistic care and provide social work services	8.1 Improve medical staff identification	8.1.1	Offer patient convenience to identify medical staff.	AR	P	7
	8.2 Put emphasis on psychological counselling	8.2.1	Provide psychological counselling service to postoperative patients.	JCI	P	7
	8.3 Protect patient privacy	8.3.1	Set up privacy protection facilities.	KTQ	P	7
	8.4 Develop social work services	8.4.1	Collaborate with social workers to provide nursing care.	AR	P	7
9. Harmonize doctor-patient relationship and reduce medical disputes	9.1 Solve medical disputes	9.1.1	Build institution to mitigate doctor-patient conflicts.	THA	P	5
	9.2 Manage patient complaints	9.2.1	Set up agency to tackle patient complaints.	THA	P	7
		9.2.2*	Establish archive to manage documents of patient complaints.	THA	S	3
		9.2.3*	Inform patients of medical risks by bulletin boards.	THA	S	3

The indicators are abbreviated. The full discerptions of indicators are available in the S1 Table.

ED, emergency department; IT, information technology; ICU, intensive care unit; NICU, newborn intensive care unit; OR, operation room; AUD, antibiotics use density.

Origin: THA, Tertiary Hospitals Accreditation; JCI, Joint Commission International; KTQ, Cooperation for Transparency and Quality in Healthcare; Added, indicator added by panelist in the first-round rating.

Type: S, structure; P, process.

*Indicator discarded in the first-round rating.

#### Panel meeting

Thirty-seven panelists attended the panel meeting and other two panelists participated by video conferencing. They gave their opinions on the new added indicators and the ones with median scores of 4–6, rephrased the description of 16 indicators to make these indicators more patient-focused and adapted for China’s context, resulting in a total of 59 indicators.

#### Second round rating

All 39 panelists responded the rating questionnaire for the 59 indicators by Email, and approved all of them (the median scores were 7, 8, 9 without disagreement). Table 2 presents these indicators with their types and median scores. The details of rating which were used to calculate median score and disagreement could be found in S1 Dataset.

**Table 2:** Results of second round rating.

Code	Indicator	Type	Median
**1.1.1**	Improve the layout of consulting rooms.	S	9
**1.1.2**	Set up adequate registration and payment windows.	S	9
**1.2.1**	Build hygiene maintenance system.	S	7
**1.2.2**	Keep toilets hygiene.	S	8
**1.2.3**	Enforce smoking ban.	S	8
**1.3.1**	Present direction signs to guide patients.	S	8
**1.3.2**	Provide safety alarm facilities.	S	7
**1.4.1**	Provide barrier-free facilities.	S	7
**1.4.2**	Offer radiation-free zones.	S	9
**2.1.1**	Increase appointment-booking rate.	P	9
**2.1.2**	Offer privilege and facilities to patients who use appointment service.	S	8
**2.1.3**	Increase the rate of appointment-booking for subsequent visit.	P	8
**2.1.4**	Increase the rate of appointment-booking for subsequent oral treatment.	P	8
**2.1.5**	Increase the rate of appointment-booking for subsequent prenatal examination.	P	9
**2.2.1**	Build referral system with secondary hospitals and (or) community health centers.	S	8
**2.3.1**	Offer time slots for examination reservation to inpatients.	P	7
**2.3.2**	Offer time slots for consulting reservation to outpatients.	P	8
**3.1.1**	Arrange adequate number of physicians to meet outpatients' need.	P	7
**3.1.2**	Provide clinical examination to ED patients in efficiency.	P	7
**3.1.3**	Provide biochemical examination and immunologic test to ED patients in efficiency.	P	9
**3.2.1**	Promote day surgery.	P	8
**3.3.1**	Connect ED treatment with pre-hospital care.	S	7
**3.3.2**	Ensure the amount of ED physicians.	S	8
**3.3.3**	Ensure the amount of ED nurses.	S	8
**3.4.1**	Open green channels in ED.	S	9
**3.4.2**	Implement triage in ED.	S	7
**4.1.1**	Provide reminder service by Apps.	S	7
**4.1.2**	Provide appointment-booking service by Apps.	S	8
**4.1.3**	Provide payment service by Apps.	S	8
**4.2.1**	Use IT to manage medical records.	S	9
**4.2.2**	Equip pharmacy with automation.	S	7
**4.3.1**	Provide facilities for self-help inquiry.	S	8
**5.1.1**	Offer admission and discharge instruction to inpatients.	S	8
**5.1.2**	Share patients' information in hospital transfer.	S	7
**5.2.1**	Seriously manage ward visiting.	S	8
**5.2.2**	Offer accompany service to handicap patients.	S	9
**5.2.3**	Offer nutrition service to improve inpatient's diet.	S	8
**5.3.1**	Follow up discharged patients according to doctors' advice.	S	7
**6.1.1**	Number of nurses in clinical nursing post.	S	7
**6.1.2**	Number of nurses to meet inpatients' need.	S	8
**6.1.3**	Number of nurses to in ICU.	S	7
**6.1.4**	Number of nurses in NICU.	S	7
**6.2.1**	Provide quality nursing care to inpatients.	P	8
**6.2.2**	Implement quality nursing care program in wards.	S	9
**7.1.1**	Mark surgical site in patients who will undergo surgery.	P	9
**7.1.2**	Promote inpatient identification.	P	9
**7.1.3**	Manage hand hygiene of medical staff.	P	9
**7.1.4**	Reduce patient falls.	S	7
**7.2.1**	Follow clinical pathways to manage inpatients.	P	8
**7.3.1**	Control frequency of antibiotic use over inpatient treatment.	P	9
**7.3.2**	Control AUD over inpatient treatment.	P	9
**7.4.1**	Release pricing information to public.	P	7
**7.4.2**	Expand pay per disease payment system.	S	8
**8.1.1**	Offer patient convenience to identify medical staff.	P	8
**8.2.1**	Provide psychological counselling service to postoperative patients.	S	7
**8.3.1**	Set up privacy protection facilities.	P	9
**8.4.1**	Collaborate with social workers to provide nursing care.	S	8
**9.1.1**	Build institution to mitigate doctor-patient conflicts.	S	7
**9.2.1**	Set up agency to tackle patient complaints.	S	8

The indicators are abbreviated. The full discerptions of indicator are available in S1 Table.

ED, emergency department; IT, information technology; ICU, intensive care unit; NICU, newborn intensive care unit; OR, operation room; AUD, antibiotics use density.

S, structure; P, process.

### Practice test

All 40 qualitative indicators were proven feasible, because their data could be collected by survey. Among 19 quantitative indicators, data of 13 indicators could be abstracted from hospital administrative data, while the other 6 were found unfeasible in the practice test.

Specifically, indicators 2.1.3, 2.1.4 and 2.1.5 (presented in Table 1), which were used to measure the volume of appointment service in subsequent visit, were discarded, because hospital information systems could not discriminate appointment for subsequent visit from total appointment records.

Other three unfeasible indicators were kept by adjusting the data collection methods. Indicators 3.1.2 and 3.1.3 were aimed at measuring the efficiency of examination reporting in emergency department, and required the experts to collect information of time interval from sampling to reporting the results of examinations. However, those data were not captured by information systems. In consideration of the workload in evaluation, the experts suggested to choose first ten examination reports in the morning, note their time interval in the questionnaire as the original data, and calculate an average. In the practice test, the experts also found that they failed to abstract data to calculate the percentage of discharges who have received follow-up service for indicator 5.3.1, because the medical record systems were incapable to search and sort records by follow-up advices. Therefore, they suggested to conveniently sample five discharge medical records with follow-up advices for the past three months and check whether the discharges have received follow-up service. The experts in future evaluation will be asked to note the results or numerical value of these three revised indicators in the questionnaire, and the patient information will not be exposed.

The final version of these indicators with their description is available in S1 Table.

## Discussion

In this study, we developed 56 indicators to evaluate the implementation of the Initiative, a nation-wide program aimed at improving patient experience in public hospitals. A modified Delphi technique and a practice test including both general and special tertiary hospitals were used to generate a set of indicators with face validity, utility and feasibility.

Our study has several strengths. Firstly, different from the traditional input-based structure indicator measurement (e.g. the financial investment and the number of beds or physicians), and the clinical outcome indicator measurement (e.g. mortality and complications) for China’s public hospitals [31, 32], our study generated a set of structure and process indicators heavily relevant to hospitals’ performance in improving patient experience. Some structure indicators measuring the institutional backgrounds for humanistic care and doctor-patient relationships, layout and barrier-free facilities for patient convenience were developed; some process indicators reflecting the efficiency of medical services, such as the percentage of daytime surgeries, percentage of time slots reservation were newly generated by this study. This set of indicators can comprehensively measure the quality of PCC, and will be used in the evaluation of tertiary hospitals nation-wide. These indicators enrich the quality indicator systems for monitoring public hospitals and demonstrate that the China’s government not only makes efforts to improve quality of clinical treatment, but also puts emphasis on promoting hospitals to provide health services in a more patient-focused way.

Second, our study used a modified Delphi technique (the RAND/UCLA appropriateness method) as the consensus method [20], which has been seldom used by Chinese researchers to develop quality indicators [33–35]. Seriously following the manual of the appropriateness method, we selected panelists with multidisciplinary backgrounds and geographical diversity, which could improve the applicability of this set of indicators among diverse hospitals and areas. In comparison with other expert consultation methods, such as expert interview and focus group discussion used in similar studies [15–17], the anonymous rating by Email in our study gave panelists adequate time to independently evaluate candidate indicators, and the structural panel meeting allowed panelists to express their opinions with minimum impact from talkative or authoritative panelists, which could come down to more reliable results [36].

Third, this set of indicators is feasible in both general and special tertiary hospitals when assessing the quality of PCC. This study indicates that the practice test is necessary to ensure the feasibility of indicators especially when the indicators are supposed to apply in a relative complex context [21].

This study has a number of limitations. First, we did not develop outcome indicators in this study. Ideally, proper organizational structure and appropriate process of service delivery will cause a higher probability of achieving positive outcomes. Panel review in the Delphi technique ensured the face validity of these indicators, while the construct validity of these indicators needs to be demonstrated by correlation relationship between outcome indicators and structure or process indicators [18, 37]. Therefore, indicator measurement for the Initiative implementation could be completed by the development of outcome indicators, and the construct validity of these indicators could be tested in further studies [19].

Second, limited by time and energy, we only selected indicators from three quality measurement systems which could meet the aim of the research and were familiar to us. The further study could review other indicator systems in different countries to refine the set of indicators developed in this study.

Third, this set of indicators cannot be used to demonstrate performance of individual hospital and hospital ranking. Positive or negative results of qualitative indictors, and numerical value of quantitative indicators will be obtained in the evaluation. Overall performance of those tertiary hospitals in each indicator will be calculated and reported to the MoH, which presents a panorama of the Initiative implementation. Subsequent studies could determine weighing and scoring rules for each indicator, depending on the baseline data collected in the first-year evaluation. The refined indicator system would allow for comparison of performance among different hospitals.

Fourth, data collection for some indicators are expensive or limited by current information systems. Data collection for 37 indicators depends on survey. This method is useful to obtain specific and detailed information for some indicators, and conveniently sampling is also a common method used by international hospital accreditation organizations such as JCI and KTQ [25, 26]. However, it is costly to conduct and cause financial burden in health administrations, which makes continuous monitoring of hospital performance less likely. Moreover, data for some indicator measurement cannot not captured in hospital information system. Considering the cost of data collection, experts suggested to reject some indicators, or abstract indicator data by convenient sampling with a small sample size, which may influence the validity of evaluation results [38, 39]. These limitations could be incrementally surmounted in along with the refinement of indicator system or the development of information systems, which may provide a more effective way of data collection for some indicators [40].

In conclusion, we developed a set of 56 structure and process indicators to evaluate tertiary hospitals’ performance in the implementation of the Healthcare Improvement Initiative, a national program aimed to improve quality of PCC and patient experience. The modified Delphi technique ensured the face validity of these indicators. The practice test played an important role in verifying the feasibility of data resource and data collection methods for these indicators. This set of indicators will be used in the evaluation of tertiary hospitals nation-wide, present a panorama of the quality of PCC in both general and special hospitals and inform health administrators of the ways to attain the goal of the Initiative. Further studies could be conducted to complete and refine this indicator system, determine the weighing and score for each indicator according to the baseline evaluation data, which allows for comparison of performance among different hospitals.

## Supporting information

S1 Dataset Results of two rounds of rating in panel review. (XLSX)

S1 Table Final version of structure and process indicators for monitoring the implementation of the Healthcare Improvement Initiative in tertiary hospitals. (PDF)
