# Supplementary material for: The development of indicator measure for monitoring the quality of patient-centered care in China’s tertiary hospitals
Source: PLoS One. 2018 Oct 11;13(10):e0205489. doi: 10.1371/journal.pone.0205489 (PMC6181381; doi:10.1371/journal.pone.0205489)
Supplement: S1 Table — (PDF) [file pone.0205489.s002.pdf]

S1 Table. Final version of structure and process indicators for monitoring the implementation of the Healthcare Improvement Initiative in tertiary hospitals.

Objective	Action	Indicator		Type	Description	Data collection Method
1. Optimize the layout of the facilities and build a friendly service environment	1.1 Improve the layout of clinic	1.1.1	The hospital should improve the layout of consulting rooms according to patient flow.	S	Outpatient flow analysis, based on which the consulting rooms are arranged, is completed including four parts: purpose, methods, results and implications.	S
		1.1.2	The hospital should set up adequate registration and payment windows.	S	Registration and payment windows are available in each floor of clinic building.	S
	1.2 Maintain a tidy environment	1.2.1	The hospital should build hygiene maintenance system.	S	Hygiene patrol and maintenance diary is available.	A
		1.2.2	The hospital should keep toilets hygiene.	S	Conveniently inspect a toilet in clinic building. This toilet is clean, odorless and equipped with antiskid surface, and each urinal in this toilet has been flushed and available for use.	S
		1.2.3	The hospital should be non-smoking.	S	There are non-smoking signs in hospital gate and entrance of clinic building. Conveniently inspect a floor of clinic building, there are non-smoking signs in each waiting area and the staircase. No butt is found in the process of survey.	S
	1.3 Construct cautionary infrastructure	1.3.1	The hospital should present direction signs to guide patients.	S	Architectural plan and department location illustration are presented in each floor of clinic building.	S
		1.3.2	The hospital should provide safety alarm facilities.	S	There are warnings of radioactive source in front of each radiological examination room.	S
	1.4 Construct user-friendly public facilities	1.4.1	The hospital should provide barrier-free facilities.	S	At least four of following six facilities are available in the hall of clinic building: terminal for appointment, registration and inquiry, water dispenser, ED telephone, wheel-chair, cart, and paper and pen.	S

		1.4.2	The hospital should offer radiation-free zones.	S	Conveniently inspect a radiological examination room, changing area, protective clothing and protective door and (or) window are available.	S
2. Promote utilization of medical appointment services and guide patient flow	2.1 Promote appointment-booking service	2.1.1	The hospital should increase appointment-booking rate.	S	Percentage of patients who use appointment service. Numerator: the number of outpatients who visited doctors by appointment; denominator: total number of outpatients.	A
		2.1.2	The hospital should offer privilege and facilities to patients who use appointment service.	S	Registration system gives visiting priority to outpatients who use appointment, and the hospital provides patients with at least four of following five facilities to make appointment: online application, telephone reservation, clinic room reservation, window reservation and community health center registration.	S
	2.2 Push forth dual-referrals	2.2.1	The hospital should build referral system with secondary hospitals and (or) community health centers.	S	Referral protocol (s) or contract(s) has (have) been signed.	S
	2.3 Allocate time slots for reservation	2.3.1	The hospital should offer time slots for examination reservation to inpatients.	S	Percentage of inpatients who reserved examinations in time slots. Numerator: the number of inpatients who reserved examinations in time slots; denominator: total number of inpatients who reserved examination.	A
		2.3.2	The hospital should offer time slots for consulting reservation to outpatients.	S	Percentage of outpatients who reserved consulting in time slots. Numerator: the number of outpatients who reserved consulting in time slots; denominator: total number of outpatients who reserved consulting.	A
3. Improve service efficiency by rational allocation of resources	3.1 Appropriately distribute resource	3.1.1	The hospital should arrange adequate number of physicians to meet outpatients' need.	S	Average number of outpatients received by each physician. Numerator: Total number of outpatients; denominator: total number of physicians in service.	A
		3.1.2	The hospital should provide clinical examination to ED patients in efficiency.	S	Average time interval (in minute) from sampling to reporting result of first ten clinical examinations of the day.	R

		3.1.3	The hospital should provide biochemical examination and immunologic test to ED patients in efficiency.	S	Average time interval (in minute) from sampling to reporting result of first ten lab examinations of the day.	R
	3.2 Push forth day surgery	3.2.1	The hospital should promote day surgery.	S	Percentage of day surgeries. Numerator: the number of day surgeries; denominator: total number of surgeries.	A
	3.3 Bolster emergency department staffing	3.3.1	The hospital should connect ED treatment with pre-hospital care.	S	Documents of regulation and information system to share patients' conditions between physicians of ED and pre-hospital are available.	S
		3.3.2	The hospital should ensure the amount of ED physicians.	S	Percentage of regular ED physicians. Numerator: the number of regular ED physicians; denominator: total number of ED physicians.	A
		3.3.3	The hospital should ensure the amount of ED nurses.	S	Percentage of regular ED nurses. Numerator: the number of regular ED nurses; denominator: total number of ED nurses.	A
	3.4 Improve treatment of the critical ill	3.4.1	The hospital should open green channels in ED.	S	Documents of implementation strategy are available. There is ambulance stop in front of resuscitation room. Ultrasonic examination room, X-ray examination room, chemical laboratory, payment windows and pharmacy are available in ED.	S
		3.4.2	The hospital should implement triage in ED.	S	Documents of red-yellow-green triage institution are available. Conveniently inquire one of ED nurses (whom work shifts to be the triage nurse in China's hospitals). This nurse can elaborate on principles to distribute patients into red areas (resuscitation room and ICU), yellow areas (observation room for critical cases), and green areas (consulting room and treatment room).	S
4. Improve service efficiency by	4.1 Strengthen information guidance	4.1.1	The hospital should provide reminder service by Apps.	S	At least two of following three Apps are available for reminder service: microblog, WeChat (a chat software popular in China), and hospital website.	S

rational allocation of resources		4.1.2	The hospital should provide appointment-booking service by Apps.	S	At least two of following three Apps are available for appointment-booking service: microblog, WeChat, and hospital website.	S
		4.1.3	The hospital should provide payment service by Apps.	S	Applications for mobile payment and one-stop payment are available in website.	S
	4.2 Strengthen information management	4.2.1	The hospital should use IT to manage medical records.	S	Information systems are used to manage medical records for both inpatients and outpatients.	S
		4.2.2	The hospital should equip pharmacy with automation.	S	Automation equipment which can use robotic manipulators to dispense drugs and transfer drugs by slideways, is available in outpatient pharmacy.	S
	4.3 Promote inquiry service	4.3.1	The hospital should provide facilities for self-help inquiry.	S	Electronic inquiry facilities with printing function, which can be used to inquire about visiting information and results of examination, are available in the hall of clinic building.	S
5. Improve process reengineering and accommodation in inpatient department	5.1 Enhance hospitalization process	5.1.1	The hospital should offer admission and discharge instructions to patients.	S	Conveniently inspect a consulting room in outpatient department and a nurse station in impatient department, printed instructions for admission and discharge are available.	S
		5.1.2	The hospital should share patients' information in hospital transfer.	S	There is a full-time office (e.g. the outpatient service office or the medical records room) to copy medical records for transferred patients.	S
	5.2 Improve hospital living conditions	5.2.1	The hospital should seriously manage ward visit and accompany to build a quiet and safety hospitalization environment.	S	Document of stipulation are available. Indicator boards of visiting time and number of accompany are available at every entrance of impatient department. There are security men to guard every entrance in non-visiting time.	S
		5.2.2	The hospital should offer accompany service to crippled patients when they take examination.	S	Documents of regulation are available. Nursing rota includes job arrangement about accompany service.	S
		5.2.3	The hospital should offer nutrition service to improve inpatient's diet.	S	There is nutrition department with nutritionist (s) of professional qualifications in the hospital.	S

	5.3 Develop patient follow-up	5.3.1	The hospital should follow up discharged patients according to doctors' advice.	S	Conveniently sample five discharge medical records with follow-up advices for past three months, at least four of which have follow-up records.	M
6. Continuously improve quality of nursing care and enhance nursing workforce	6.1 Bolster nursing staff	6.1.1	The hospital should ensure the number of nurses in clinical nursing post.	S	Percentage of nurses in clinical nursing post. Numerator: the number of nurses in clinical nursing post; denominator: total number of nurses.	A
		6.1.2	The hospital should ensure the number of nurses to meet inpatients' need.	S	Ratio of nursing staff to number of beds in inpatient department.	A
		6.1.3	The hospital should ensure the number of nurses to meet ICU patients' need.	S	Ratio of nursing staff to number of beds in ICU.	A
		6.1.4	The hospital should ensure the number of nurses to meet NICU patients' need.	P	Ratio of nursing staff to number of beds in NICU.	A
	6.2 Consolidate quality care	6.2.1	The hospital should provide quality nursing care to inpatients.	P	Ratio of wards involved in quality nursing care program to entire wards.	A
		6.2.2	The hospital should implement quality nursing care program in wards.	P	Documents of regulation and implementation strategy are available. Conveniently inquire five nurses in impatient department, at least three of which are aware of the content of eleven components of the quality nursing care program (including admission nursing, morning nursing, evening care, diet nursing, excretion nursing, supine care, comfortable nursing, preoperative care, postoperative care, patient safety management and discharge nursing).	S
7. Ensure patient safety by adoption of standard	7.1 Consolidate patient safety	7.1.1	The OR should mark surgical site in patients who will undergo surgery.	P	Conveniently sample five postoperative medical records and inspect the operational safety checklists (including patient information, surgical method and surgical site etc.). Each checklist has been completed and signed by the patient, anesthetist and surgeon.	S

operating procedures		7.1.2	The hospital should promote identity recognition of inpatients.	P	Documents of regulation and procedures of inpatient identity recognition before sampling, drug delivery and treatment are available. The tags of samples and drugs include at least two of the following ways to identify patients: name, age (or data of birth), medical record number and bed number.	S
		7.1.3	The hospital should manage hand hygiene of physicians and nurses.	P	Documents of regulation and training records are available. Conveniently sample a physician and a nurse in outpatient department and inpatient department respectively. They can correctly demonstrate six-step scrubbing method.	S
		7.1.4	The hospital should prevent patients fall.	P	Documents of implementation strategy for fall prevention are available. There are handrails in corridors of clinic building and inpatient department. Wards are equipped with night lights and beds with side rails.	S
	7.2 Develop clinical pathways	7.2.1	The hospital should follow clinical pathways to manage inpatients.	P	Percentage of discharges who were managed in clinical passway. Numerator: the number of discharges outpatients who were managed in clinical passway; denominator: total number of discharges.	A
	7.3 Strengthen appropriate medication	7.3.1	The hospital should control frequency of antibiotic use over inpatient treatment.	P	Percentage of inpatients who used antibiotic. Numerator: the number of inpatients who used antibiotic; denominator: total number of inpatients.	A
		7.3.2	The hospital should control AUD over inpatient treatment.	P	The AUD is less than 40DDDs per 100 inpatients.	A
	7.4 provide transparent charge service	7.4.1	The hospital should release pricing information to public.	P	Pricing information is available in boards in visiting hall and (or) in hospital website.	S
		7.4.2	The hospital should expand pay per disease payment system.	P	The hospital includes more diseases in pay per disease payment system than last year.	A

8. Strengthen humanistic care and provide social work services	8.1 Improve medical staff identification	8.1.1	The hospital should offer patient convenience to identify medical staff.	P	Conveniently sample five physician and (or) nurses, all of which wear chest cards without shelter or indistinctness of their name.	S
	8.2 Put emphasis on psychological counselling	8.2.1	The hospital should provide psychological counselling service to postoperative patients.	P	Documents of institution are available. Conveniently inquire five postoperative patients, three of which have received counselling service.	S
	8.3 Protect patient privacy	8.3.1	The hospital should set up privacy protection facilities.	P	There are screens in wards with multiple beds, and diagnosis field is excluded from bedside card.	S
	8.4 Develop social work services	8.4.1	The hospital should collaborate with social workers to provide nursing care.	P	There are social workers in service.	S
9. Harmonize doctor-patient relationship and reduce medical disputes	9.1 Build institution to solve medical disputes	9.1.1	The hospital should build institution of mediations and corresponding insurance payments to mitigate doctor-patient conflicts.	P	Documents of institution are available. There are records of medication and (or) insurance claims.	S
	9.2 Manage patient complaints	9.2.1	The hospital should set up specialized agency to tackle patient complaints.	P	Outpatient office and full-time staff is available in the hospital. There are records of feedback to patient complaints.	S

ED, emergency department; IT, information technology; ICU, intensive care unit; NICU, newborn intensive care unit; OR, operation room; AUD, antibiotics use density.

Type: S, structure; P, process.

Data collection method: S, survey; A, administrative data; M, medical records; R, examination reports.
